# Quality Management and Sustainability in the Design of Active Biocomposites: Evaluation of Double-Layer Protein–Polysaccharide Complexes Enriched with Plant Extracts

**DOI:** 10.3390/molecules30214259

**Published:** 2025-10-31

**Authors:** Nikola Nowak-Nazarkiewicz, Wiktoria Grzebieniarz, Joanna Tkaczewska, Agnieszka Cholewa-Wójcik, Michał Kopeć, Krzysztof Gondek, Hanna Derechowska, Ewelina Jamróz

**Affiliations:** 1 Department of Chemistry, University of Agriculture, Balicka 122, 30-149 Kraków, Poland; 2 Department of Animal Product Technology, Faculty of Food Technology, University of Agriculture, Balicka 122, 30-149 Kraków, Poland; 3 Department of Packaging and Logistics Processes, Cracow University of Economics, Rakowicka 27, 31-510 Kraków, Poland; 4 Department of Agricultural and Environmental Chemistry, Faculty of Agriculture and Economics, University of Agriculture, Aleja Mickiewicza 21, 30-149 Kraków, Poland

**Keywords:** active biocomposites, biodegradable films, film quality management

## Abstract

Despite promising results, biocomposite research still requires elaboration, particularly with regard to functional properties and applications. In this study, multilayer biocomposites based on gelatin, κ-carrageenan and carboxymethylcellulose were enriched with sage or blackberry extracts. The films were characterized based on their physicochemical traits and bioactivity for application as active packaging and environmental biodegradation. FTIR confirmed extract integration and strong matrix interactions, while UV-VIS analysis showed efficient UV blocking. Water properties remained acceptable (WVTR ≈ 550 g/m^2^ × d); solubility decreased for BB (41.73% vs. 53.45% control). Mechanical testing indicated a plasticizing effect: elongation increased (20.00% control; 35.35% BB; 39.29% SAGE), while tensile strength and Young’s modulus decreased. Antioxidant capacity rose (FRAP: 0.38 control, 1.97 BB, 4.48 SAGE µTrolox/mg; DPPH: 6.38% control, 85.68% BB, 78.25% SAGE; MCA: none). During refrigerated storage, antimicrobial effects were most evident on days 6–9. Lipid oxidation peaked for BB (0.92 mg MDA/kg, day 9), while pH was more stable with SAGE. Biodegradation and phytotoxicity confirmed environmental safety and compostability, with increased humic acid carbon in vermicompost. Overall, the results confirm the relevance of modifying biopolymers using green chemistry and highlight their importance for quality management, food safety and sustainable circular economy strategies.

## 1. Introduction

The development of innovative packaging materials is gaining increasing importance in the search for alternatives to petroleum-based plastics, aligning with the circular economy paradigm and the implementation of sustainable development strategies. The adoption of active biopolymer composites should therefore be examined not only in terms of their physicochemical properties but also from the perspective of quality management, food safety assurance and efficiency of production and logistics processes [1,2,3].

Therefore, this study aims to develop and evaluate innovative biocomposite films based on proteins and polysaccharides, enriched with natural plant extracts (sage and blackberry) as active and sustainable packaging materials. The research focuses on establishing the relationship between the composition of the biopolymer matrix and its structural, mechanical and barrier properties, as well as on evaluating the impact of bioactive components on the antioxidant and antibacterial activity of the film. In particular, it analyses the impact of proteins and polysaccharides, which may determine the functionality and stability of the composites obtained.

The study also aims to verify the suitability of the developed materials in real food packaging systems by assessing their impact on the quality and microbiological stability of Atlantic salmon fillets. The results obtained may be used to support active materials, especially biodegradable packaging materials that comply with the principles of sustainable development and quality management.

Biopolymer composites can essentially replace petrochemical-based materials, especially in the context of single-use plastics, including food packaging. The ability of packaging material to protect the product depends to a great degree on its physical properties, and on its structure and molecular interactions as well. The interactions between biopolymers forming the matrix, as well as the functionalization of biocomposites with active ingredients, are areas of fundamental significance [4,5]. At the current level of knowledge, the priority action in filling the knowledge gap is the method of modifying natural composites [6,7]. The functional properties of biopolymer materials differ from those of conventionally used materials and should be modified, but research should additionally be focused on exploiting the characteristics and properties of biopolymer materials resulting from their origin and particle characteristics. Apart from their undeniable advantage over conventional petroleum-based materials, which is their environmental non-toxicity and easy biodegradability, biopolymers are also distinguished by their particular modifiability, especially in the field of green chemistry [8].

In this context, one of the most promising approaches is to exploit the individual natural properties of biopolymers, which spontaneously exhibit binding and supporting assets. Protein-based materials exhibit better barrier properties than those based on pure polysaccharides, while polysaccharides, in turn, exhibit better mechanical properties [9,10]. The combination of these environmentally safe and promising macromolecular compounds, as well as the modification of the matrix, seems to be a promising direction in the modification and design of new biopolymers. Such mixtures and complexes can form matrices of varying polarity and water accessibility, which can affect the stability and release of the implemented bioactive molecules. The structural stability of biopolymer composites primarily results from non-covalent interactions, such as hydrogen bonds and electrostatic forces between complementary functional groups. These enable the formation of a three-dimensional, ordered network. The synergistic effect between the matrix components depends on their chemical compatibility and, in particular, on the presence of complementary functional groups, which may consequently contribute to the formation of more ordered structures with physicochemical properties that differ from the original ones [11,12].

An equally important direction is the incorporation of bioactive plant-based ingredients. Extracts and essential oils are rich in compounds such as polyphenols, flavonoids, tannins and anthocyanins, and they exhibit antioxidant and antimicrobial activity [13,14]. Their effective integration into the biopolymer matrix can improve the internal structure of the composite and significantly enhance its functional properties (Dirpan et al., 2023; Dutta & Sit, 2023 [15,16]). Such functionalization also contributes to innovation management and value creation within food supply chains, offering solutions consistent with the principles of green chemistry and sustainable development.

In light of the above considerations, this study is focused on the development and evaluation of double-layer protein–polysaccharide composites enriched with plant extracts (sage and blackberry). Their applicability is analyzed in the context of quality management systems and sustainable development standards. The films were comprehensively characterized in terms of chemical structure (Fourier-Transform Infrared spectroscopy—FTIR, Ultraviolet–Visible spectroscopy—UV-Vis), water vapor permeability, mechanical properties and water-related characteristics. Their applicative potential was assessed through packaging trials with Atlantic salmon fillets, including antioxidant, microbiological and physicochemical evaluations. Finally, the environmental performance of the materials was analyzed through biodegradability, phytotoxicity and humic substance transformation studies. This integrated approach enables an assessment of the composites not only as functional packaging materials but also as tools supporting quality management, sustainability and circular economy strategies [3,15,16].

This study builds upon current knowledge in the field of green chemistry and existing research gaps are addressed by developing multilayer materials containing a protein–polysaccharide complex enriched with natural-based active compounds, with particular emphasis on their potential contribution to quality management, food safety and sustainability standards. A double-layer material was developed, the first layer of which consisted of a gelatin and κ-carrageenan complex enriched with biologically active plant extracts: sage and blackberry extracts. The second layer consisted of carboxymethylcellulose. The biocomposites were then subjected to a comprehensive analysis of their chemical properties (chemical structure by FTIR, properties towards UV light using UV spectroscopy), as well as water vapor barrier, mechanical and water properties. The material was then subjected to extensive analysis of its potential for use as a packaging material for Atlantic salmon fillets by assessing its antioxidant activity using 2,2-Diphenyl-1-picrylhydrazyl (DPPH) and Ferric Reducing Antioxidant Power (FRAP) methods and its ability to chelate metal ions. Next, the microbiological purity of the stored salmon, the degree of lipid oxidation and the pH of the samples were assessed. In the final step, in order to perform a comprehensive analysis of the developed composites, their impact on the natural environment was assessed by evaluating their effect on cress growth (*Lepidium sativum* L.), biodegradability and analysis of humic substances in vermicompost. This represents a comprehensive approach, combining material modification and multi-level analysis, starting from the formation of the protein–polysaccharide complex, through the development and characterization of the biocomposite, on to the evaluation of its environmental impact. In this way, the full life cycle of the material was taken into account.

## 2. Results and Discussion

### 2.1. Physicochemical and Structural Characterization of the Films

**Figure 1:**
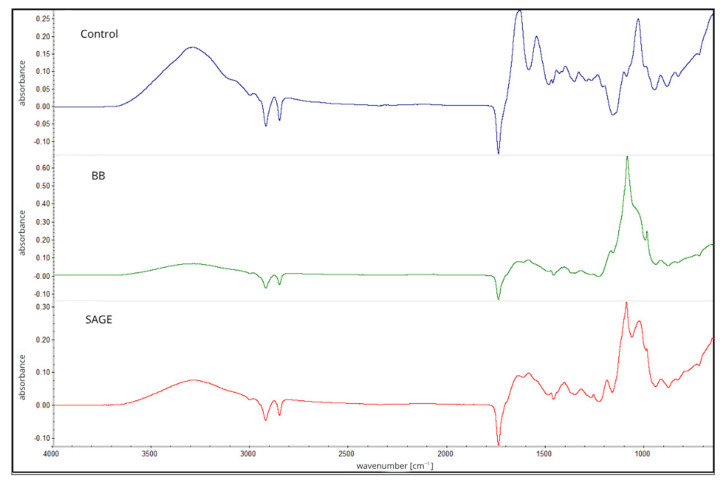
FTIR spectra of first layer of developed films, composed of carrageenan and gelatin without extracts (Control) or with addition of either blackberry fruit (BB) or sage flower (SAGE) extracts.

Polysaccharides and proteins, which can be used as chemical compounds for forming biopolymer films, are characterized by diverse physicochemical properties. The combination of gelatin, carrageenan and carboxymethylcellulose makes it possible to obtain a matrix that combines the very good film-forming properties of polysaccharides with the low gas permeability exhibited by proteins. The development of complexes or mixing polysaccharide and protein solutions can lead to the formation of stable networks between biopolymers through hydrogen bonds or electrostatic interactions [17]. In addition, the biopolymer matrix should also be suitable for the incorporation of active ingredients, especially in the case of plant extracts, which introduce additional functional groups such as hydroxyl, carboxyl or aromatic groups that can interact with polymer chains [18]. In this paper, the structures of the compounds and the effectiveness of the extracts were analyzed using FTIR (Figure 1).

FTIR spectra of the first layer (Figure 1), composed of carrageenan and gelatin (control) with the addition of either sage flower extract (SAGE) or blackberry fruit extract (BB), were subjected to analysis. In all of the analyzed composites, a broad band associated with the stretching of –OH groups and amide A characteristic of gelatin within the range of 3390–3450 cm^−1^ can be observed [19]. The intensity of this band is highest in the control film, which may indicate the effective implementation of active ingredients into the biopolymer matrix and a reduction in the availability of free –OH and N–H groups [20].

The bands observed within the range of 2850–3000 cm^−1^ are attributed to C–H and CH_2_ stretching vibrations, including amide III. Absorption bands in the range of 1220–1230 cm^−1^ and at 1034 cm^−1^ correspond to sulfate ester groups (-SO_3_^-^), which are characteristic of κ-carrageenan. Additionally, the bands between 1030 and 1060 cm^−1^ are associated with the stretching vibrations of C–O–C glycosidic linkages [21,22].

In this layer, changes in IR spectra were also observed at the wavelength range of approximately 1510 cm^−1^, which are caused by the presence of aromatic compounds in plant extracts. The lower intensity in films with extracts (BB, SAGE) indicates that these groups are more strongly bound with phenols by hydrogen bonds and other active compounds [23].

**Figure 2:**
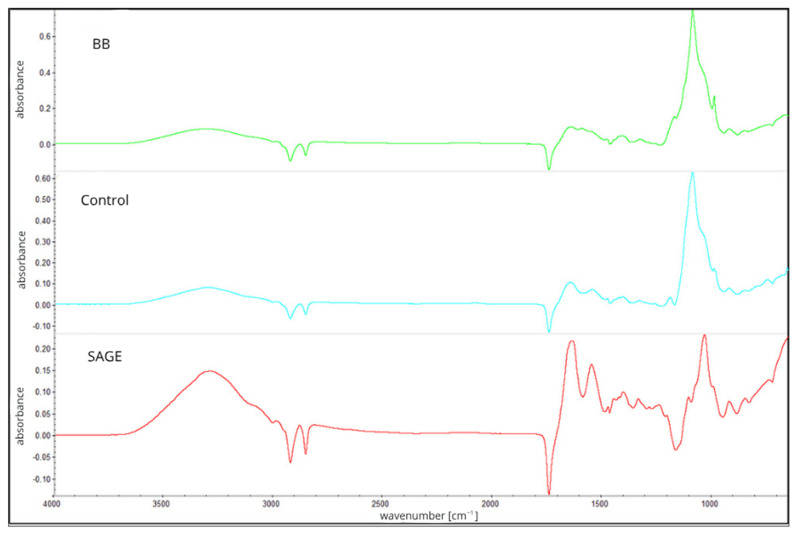
FTIR spectra of CMC-based second film layer without extracts (Control) or containing blackberry (BB) or sage (SAGE) extracts.

Analysis regarding the spectrum of the second layer (Figure 2), which consisted of carboxymethylcellulose, showed characteristic vibrations at 2900 cm^−1^ which are connected with aliphatic C-H, typical for polysaccharides, and strong vibrations between 1580 and 1600 cm^−1^, which are associated with the asymmetric stretching vibrations of ionized carboxylate groups (COO−), confirming the presence of carboxymethyl substituents in the cellulose backbone. Additional intense bands within the range of 1030–1100 cm^−1^ were attributed to C–O and C–O–C stretching vibrations within the glucopyranose units of the cellulose chain. The presence and consistency of these bands across all film types (control, SAGE, BB) suggest that the addition of plant extracts in the lower layer had no significant chemical impact on the structure of the upper CMC layer [24,25].

The thickness of the film was also measured, which revealed statistically significant differences between all samples, with the highest value recorded for the BB film (0.0404). Film thickness can affect the intensity and shape of bands in FTIR spectra, leading to increased absorbance or band distortion [26]. It is important to note, nonetheless, that the addition of plant extracts may also affect the structure of the biopolymer network. This phenomenon may result from interactions between phenolic compounds contained in the extracts and functional groups of polymer chains, leading to changes in molecular order and, consequently, to an increase in the thickness of the films obtained [27,28]. All analyzed films contained the same amount of dry biopolymer mass, differing only in the presence and type of active ingredient, which indicates that the observed differences in thickness are mainly due to modifications in the biopolymer structure under the influence of the addition of extracts, rather than changes in its concentration.

The slight negative peaks observed near 2900 cm^−1^ and 1750 cm^−1^ could be related to subtle differences in baseline correction and plasticizer distribution in the film matrix. Glycerol, used as a plasticizer, exhibits characteristic absorption bands in these areas, and its uneven distribution or molecular interactions with the biopolymer network may slightly affect the spectra obtained [29].

It should also be noted that such negative bands may sometimes result from instrumental or technical factors, such as excessive background compensation, slight differences in sample thickness, or contamination of the ATR crystal during background collection [26].

**Figure 3:**
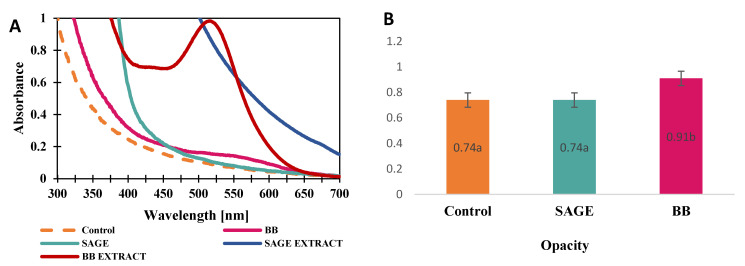
(**A**) UV–Vis spectra of developed films within the range of 300–700 nm; (**B**) Opacity values of films. Identical letters (a,b) indicate no statistically significant differences between samples.

The mechanism of UV light absorption depends on the type of biopolymers used in the design of UV protective films/coatings. Most biopolymers contain a basic skeleton of repeating carbohydrate units that include amino, hydroxyl, alcohol, acid, ketone and aldehyde functional groups. The absence of aromatic structures results in a significantly lower probability of π-π transition, and therefore absorption is difficult or very weak in the UV range [30]. Therefore, films based on biopolymers alone can transmit all UV, visible and near-infrared radiation during exposure. As a consequence, their original properties are altered and the internal components of the packaging materials deteriorate, weakening their original strength and functionality. UV rays, which have a greatly adverse effect, are mainly divided into ultraviolet A (UV-A), ultraviolet B (UV-B), and ultraviolet C (UV-C). Among them, UV-A radiation, with a wavelength of 320–400 nm, accounts for 95% of the radiation reaching human skin. UV-B radiation has a wavelength of 280–320 nm and causes degradation of the internal components of a packaging material, reducing its performance and thus shortening its shelf-life. The UV protection properties of packaging materials can be modulated by adding active ingredients with UV absorption properties (200–400 nm) [31,32]. The ability of the developed composites to block UV light and their level of opacity are shown in Figure 3.

The UV blocking properties are synonymous with the degree of the materials’ opacity. The highest UV blocking capacity within the wavelength range of 475–625 nm was observed for the BB film, similarly to which the highest degree of opacity (0.91) was observed. The degrees of opacity for SAGE and the control films do not differ statistically, but the UV-Vis spectrum indicates a greater ability to block UV light by the film containing the sage extract, especially within the wavelength range of 300–500 nm. Anothocyanins, present in blackberry fruit extract, are the only class of polyphenols that can absorb light within the UV (280–400 nm) and blue light (360–500 nm) ranges. The UV-blocking properties of SAGE film are caused by rosmarinic acid, the dominant organic phenol in sage, as well as the presence of carnosic acid and carnosol. This behavior is consistent with the phenolic composition of the extracts, since rosmarinic and carnosic acids in sage mainly absorb UV radiation, while anthocyanins in blackberry extract also absorb visible wavelengths [33,34,35]. The use of plant extracts, especially those rich in polyphenols, can effectively improve the performance of biopolymer films by increasing their ability to block UV light.

### 2.2. Water Properties

Due to the presence of polar functional groups—with the ability to form bonds with water molecules (including hydroxyl groups)—in their chemical structure, biopolymer materials exhibit high water solubility and hygroscopicity. As a result, when used as packaging materials, they are often unable to provide adequate product protection due to their high water vapor permeability, solubility or ability to absorb water from the packaged product. However, these materials should be slightly permeable to water vapor and gases, as a complete lack of permeability can lead to condensation. Modifying biopolymer composites to reduce their water vapor permeability or solubility is therefore a desirable step towards improving biopolymer materials. The water properties of developed composites are presented below (Table 1) [17,36,37].

The literature contains reports in which it is noted that the addition of aqueous plant extracts increases the solubility of a material in water or its permeability to water vapor. This is most likely related to the action of phenols, which can increase the hydrophilicity of a film [38]. However, in this study, no negative effects of the plant extracts on the water properties of the film were observed. Interestingly, in the case of the blackberry extract, a significant decrease in water solubility was demonstrated (41.73% compared to 53% for the control film). This effect may result from the presence of flavones in the extract, compounds known for their moderate solubility. In addition, this decrease may also have been related to the possible interaction of the extracts with the biopolymer matrix. The phenolic compounds contained in the blackberry extract can form hydrogen bonds or π–π interactions with the gelatin and carrageenan chains, leading to sealing the structure and limiting water migration into the film [39]. FTIR spectrum analysis also indicated good integration of the extracts with the biopolymer matrix. This may be of practical importance when using the film for packaging foods with high moisture content, as reduced solubility promotes greater material stability during storage.

The water content in all of the obtained composites is approximately 10%, with the addition of sage extract increasing the water content by three percentage points compared to the control. The observed increase in water content may result from the presence of numerous hydroxyl groups in the structure of rosmarinic acid and other phenols, which increase the affinity of the material to water molecules. This value is characteristic and typical for biopolymer films [40,41].

The water vapor transmission rate for all analyzed samples is approx. 550 g/m^2^ × d. This value is within the range considered low for biopolymer materials [42]. Low water vapor permeability is an important functional advantage in packaging applications as it reduces the risk of product moisture and limits the growth of microorganisms in its environment. The obtained results indicate that the presence of aqueous plant extracts did not impair the barrier properties of the composites, and even had a beneficial effect on them.

### 2.3. Mechanical Properties

Biopolymer materials, especially with the potential for use as packaging materials, should be characterized not only by low permeability to gases but also by appropriate mechanical properties to ensure protection of products during distribution or transportation. The use of multilayer materials and protein–polysaccharide complexes is an innovative and promising step in improving the mechanical properties of biopolymer composites. The formation of complexes and layered systems can promote the formation of numerous physicochemical interactions between components and, consequently, improve mechanical properties [43]. The mechanical properties of the developed films are shown in Table 2.

The biopolymer film, prepared as a complex of gelatin and carrageenan with a second layer of CMC polysaccharide (Control), showed higher maximum breaking load and lower elasticity compared to films containing plant extracts (Table 2). The significant reduction in breaking load values for BB and SAGE films can be explained by the introduction of extract molecules into the matrix, which modifies the internal cohesion of the polymer network and affects the value of shear stress [44,45].

On the other hand, the significantly higher values of elongation at break (Table 2) for BB (35%) and SAGE (39%) compared to the control film (20%) indicate that the extracts increase the plasticity of the material, probably due to intermolecular interactions involving water and phenols—which is consistent with the observations of Pan et al. [40], noting that water acts as a natural plasticizer, increasing elongation in films based on proteins and polysaccharides. The analyzed BB and SAGE films had 1.65 and 2.78 (respectively) percent higher water content. In addition, the plasticity enhancement can be chemically attributed to the presence of phenols such as flavonoids and rosmarinic acid, among others, which can form hydrogen bonds with biopolymer chains, leading to partial reorganization of the film structure and increased chain mobility, causing higher elasticity with reduced strength [40,45,46].

As expected, the control film showed the highest value of Young’s modulus (Table 2) (477.58 MPa), indicating high stiffness due to the compact structure of gelatin and carrageenan. In contrast, films with plant extracts (SAGE and BB) exhibited significantly lower E values (103.23 MPa and 127.33 MPa, respectively), confirming the plasticizing effect caused by the presence of phenolic compounds. These compounds, particularly flavonoids and rosmarinic acid, can weaken the interactions between polymer chains, increasing their mobility and flexibility. These observations are in line with those presented in the literature, repeatedly demonstrating a decrease in Young’s modulus when plant extracts are introduced into the biopolymer matrix [47].

### 2.4. Bioactive Properties of the Films

Applicative property evaluation of the developed packaging composites began with an analysis of their antioxidant potential. The assessment was carried out using three complementary methods: the FRAP test, which assesses the reducing power of compounds by converting Fe^3+^ to Fe^2+^; the DPPH test, which measures the hydrogen atom-donating ability of the composites; and the metal chelating activity test, which determines their capacity to complex transition metal ions responsible for catalyzing lipid oxidation. The results are presented in Table 3.

The highest antioxidant properties (Table 3), which may indicate the expected active effect of the materials, were exhibited by films with the addition of active extracts. In terms of the ability to chelate Fe^3+^ metal ions, the film with the addition of sage extract had the highest value (4.48 compared to 0.38), but these values remained moderate. In the analysis of the ability to scavenge DPPH free radicals, both films with extracts (BB, SAGE) showed high values, which confirms their effectiveness as active components in the biopolymer matrix. However, none of the films demonstrated any metal ion chelating ability. The SAGE biocomposite exhibited chelating activity toward Fe^3+^ ions, whereas no chelation activity was observed for Fe^2+^ ions. This phenomenon may be related to the fact that the chelating activity of phenolic compounds strongly depends on the chemical structure of individual phenols. The interaction of phenols with iron ions depends on their chemical structure and composition, location and number of OH, SH or COOH groups [1,2]. Nevertheless, the addition of BB and SAGE extracts clearly improves the antioxidant properties of the film, which may protect packaged food from oxidative degradation. SAGE shows greater reducing ability (FRAP), while BB exhibits larger radical activity (DPPH). Perhaps the composition of the extracts, despite the presence of phenols, did not contain sufficient ligands capable of complexing transition metal ions under the test conditions. The high free radical neutralizing capacity of the active films is most likely due to the presence of flavonoids, anthocyanins and rosmarinic acid, which act as proton donors and stabilize free radicals through structural resonance [48,49,50].

Analysis of microbiological properties in vitro (Table 4) did not reveal any inhibitory effect on the growth and development of microorganisms in the group of tested organisms, with the exception of the effect of the film on the growth of *Aspergillus flavus*. Neither carrageenan nor CMC has documented antimicrobial properties; therefore, the obtained effect can be attributed to the presence of the plant extract. Data from the literature indicate that the *Salvia officinalis* extract obtained by maceration, as well as extracts and essential oils from *Lavandula officinalis*, exhibit antifungal activity against *Aspergillus flavus* strains [51].

However, it should be emphasized that the conducted studies were short-term and involved a limited period of in vitro observation. Under such conditions, there is a risk that the active compounds present in plant extracts remain partially bound in the film matrix and are released slowly. For this reason, it was important to conduct further in vivo studies and over a longer period of time to verify whether the antimicrobial activity of the film increases with incubation time and the gradual release of bioactive compounds. It was also of significance to assess its potential for use as an active packaging material.

### 2.5. Packaging Performance Evaluation

In order to assess the practical applicative potential of the developed biocomposites as packaging materials, a study was conducted involving the storage of fresh salmon fillets packaged in the analyzed films, with the LDPE film used as a control sample. Salmon fillets were used as a model product due to their high fat content and susceptibility to oxidative and microbiological processes. This raw material is particularly sensitive to storage conditions, which is why it can be a suitable model for assessing the effectiveness of packaging materials with active properties.

**Figure 4:**
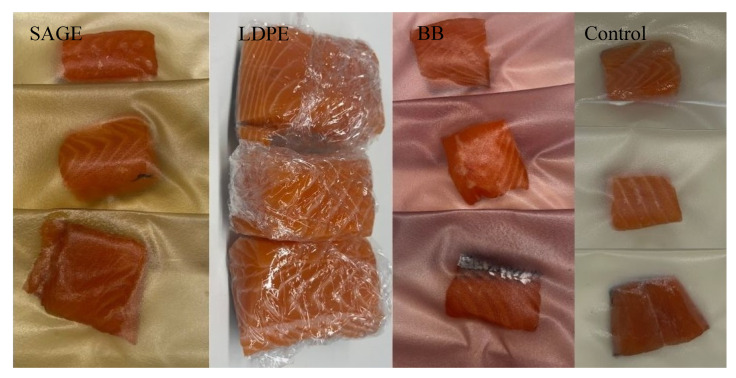
Packaging method of salmon fillets for in vivo analyses.

The packaging method is shown in Figure 4. The changes in lipid oxidation (TBARS), product pH and microflora development during cold storage were evaluated.

**Figure 5:**
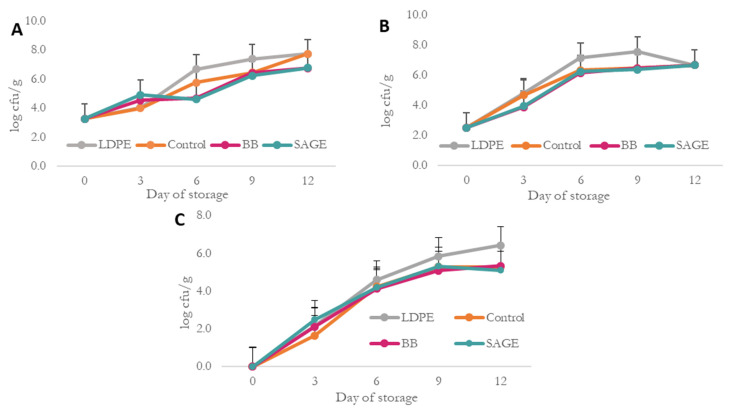
Microbiological properties of films. (**A**) total bacterial count; (**B**) psychotropic bacteria; (**C**) yeasts and molds.

The microbiological analysis allowed us to determine how the developed composites affect the growth of aerobic bacteria, psychrotrophs, yeast and mold under refrigerated conditions. It also made it possible to verify whether, despite numerous reports in the literature regarding antioxidant activity, the implementation of the developed composites in the production of fish products is justified. Moreover, it allowed us to verify whether the addition of plant extracts contributed to the development of an active composite with potential antimicrobial properties. The results of the microbiological analyses are presented in Figure 5.

The initial total microbial count (Figure 5A) on day 0 was 3.3 log CFU/g, as determined from analyses of randomly selected fresh fillet samples prior to packaging. Subsequently, on the third day of the analysis, an increase in the total number of microorganisms was observed in all samples, but for BB and SAGE, it was significantly higher than for the control samples (biopolymer and LDPE). On the following day—the sixth day of analysis—the differences became more pronounced, with the highest total microbial count observed in the sample stored in LDPE film, amounting to 6.69 log CFU/g, compared to 4.69 and 4.20 log CFU/g for BB and SGE, respectively. A similar trend was also noted on day 9, but then the values for the biopolymer control, BB and SAGE were similar, which may indicate that the slow release of antibacterial components from the active films was most intense until day 6 of the analysis. On the final day of the analysis, the biopolymer control film and LDPE showed a similar microbial count (approximately 7.70 log CFU/g), while the values for the active films were approximately 6.80 log CFU/g, which indicates the antimicrobial activity of the active composites used.

Analysis of psychrotrophic bacteria counts (Figure 5B) revealed significant differences among the active films and LDPE film between days 6 and 9 of storage. On day 6, the total number of psychrotrophic bacteria was highest in samples stored in LDPE films (7.15 log CFU/g), while in samples packed in biopolymer films, the values ranged from 7.2 to 7.3 log CFU/g. The initial value on day 0 was approximately 2.50 log CFU/g for all samples and increased throughout the storage period. On the last day of analysis, the value was similar for all samples and amounted to approximately 6.70 log CFU/g.

On the first day of analysis, the number of yeasts and molds for all samples was the same and totaled 0 (Figure 5C). A significant increase in the number of yeasts and molds was observed for all samples throughout the storage period, with the highest increase presented for samples stored in LDPE films. Differences were already observed on the 6th day of the analysis (4.61 log CFU/g for LDPE, 4.11 log CFU/g for BB), but they were not statistically significant. On the following day of analysis—the 9th day—the differences between these samples were already around 0.7 log CFU/g. On the last day of analysis, significant differences were observed between biopolymer-based films and the LDPE film, with higher results obtained for the latter.

This analysis clearly showed that the developed biocomposites exhibit antimicrobial activity and thus have the potential to be used as active food packaging. Despite their relatively high water solubility (40%), the films retained their structural integrity during contact with the product, confirming their suitability for short-term use in packaging food with high moisture content. Although biopolymer films have a higher water vapor transmission rate (WVTR) than conventional petrochemical-based materials, their antimicrobial properties can extend the shelf-life of packaged food products. The used extracts showed antimicrobial activity up to the 6th or 9th days of analysis, which may be due to the slow release of active substances from the biopolymer matrices. The biopolymer control film also showed antimicrobial activity, which may be due to the presence of κ-carrageenan (KC) in its structure, which has a large number of active functional groups, such as sulfate and hydroxyl groups, which make it rich in biological activity [52,53]. The antimicrobial properties of the sage extract are most likely connected with the presence of compounds such as rosmarinic acid and carnosol in its composition. The antibacterial activity of plant extracts, including sage, demonstrates a highly linear relationship with their total phenolic content. However, the exact mechanism of action of these compounds is not known. It is believed that these compounds cause irreversible damage to the cell wall, resulting in the death of bacterial cells [52].

Blackberry extracts are rich in antioxidants, tannins, anthocyanins and flavonoids, which exhibit antimicrobial activity against both Gram-positive and -negative bacteria. It is reported in the literature that polyphenols, primarily anthocyanins from blackberry extract, can negatively affect the metabolic activity of microorganisms. The mechanisms of action are not explained, but one assumption is that phenol dissociation occurs on the microorganism’s membrane, causing a sudden drop in pH inside the cell, which inhibits ATP synthesis. The antimicrobial activity of blackberry extracts may also be influenced by other polyphenols, such as phenolic acids and tannins, as well as organic acids. Organic acids have the ability to diffuse through the cell membrane, where dissociation occurs, causing changes in the pH of the cytoplasm, inhibiting enzymes and damaging the cellular structures of microorganisms, ultimately leading to cell death [48].

In the conducted in vitro analysis, no inhibitory effect on bacterial growth was detected. This indicates that the antimicrobial potential of blackberry extracts may be strongly dependent on the type of microorganism, concentrations and experimental conditions. It may also result from the insufficient release of active compounds from the film matrix during the relatively short incubation period. For this reason, extended or in vivo analyses seem to be necessary to confirm the antimicrobial effectiveness of the tested composites.

In the next step of evaluating the applicability of the developed biocomposites as active materials, their effect on packaged salmon samples was assessed by determining the amount of secondary lipid oxidation products and the pH of the samples (Table 5).

The initial TBARS value (mg MDA/kg) in the fresh fillets (day 0) was 0.08 ± 0.03, and it was determined based on analyses performed for randomly selected samples prior to packaging. The highest level of oxidation occurred in samples packaged in the BB film, reaching 0.92 ± 0.19 on day 9, which may suggest the pro-oxidative effect of certain compounds present in blackberry extract, such as anthocyanins, which, under given conditions, may generate reactive oxygen species or may not work effectively in the presence of fish lipids. The SAGE film showed more stable results, and TBARS values did not differ significantly from the control (biopolymer film without additives). LDPE film, as the reference conventional material, showed the lowest TBARS values throughout the storage period, while biopolymer films accelerated lipid oxidation [54].

The initial pH value was 6.34 ± 0.06. As storage progressed, slight pH fluctuations were observed in the BB and SAGE film samples, with the smallest range of changes in SAGE, which may indicate partial inhibition of proteolytic and microbiological processes. In the samples covered with the control film (biopolymer without extract), the pH slightly increased to 6.60 on day 9 and then decreased, which may be related to the initial decomposition of proteins, followed by fermentation and acidification by microflora [55]. In the case of the LDPE film, a clear increase in pH to 6.73 ± 0.04 was observed on day 12, which may indicate the accumulation of volatile alkaline nitrogen compounds under conditions of limited gas exchange. This suggests that although the LDPE film is effective in protection against oxidation, it may limit gas exchange, creating conditions conducive to undesirable metabolic changes.

### 2.6. Assessment of Environmental Impact

Materials based on natural compounds are presented as viable substitutes for petrochemical-based materials, which are also environmentally safe due to their origin and biodegradability. However, the assumption of biodegradability should not exempt biopolymer-based materials from testing. In numerous studies, it is emphasized that the environmental fate and degradation kinetics of natural materials depend not only on their chemical composition, but also on the processing method, with particular stress on modification [3,56]. Analysis of the potential environmental impact of the developed composites is presented in Table 6.

The biodegradation test of the developed composites showed that all analyzed films undergo effective decomposition under composting conditions. The total respiratory activity ranged from 137 to 156 mg O_2_/g dry weight during the 28-day experimental period, which indicates active degradation by microorganisms. The highest value was recorded for the film with the sage extract (SAGE: 156.3 ± 10.1 mg O_2_/g), potentially suggesting slightly better matrix accessibility for microflora, possibly due to a change in the biopolymer network structure after the addition of the extract. As shown in Table 1, this film also exhibited the highest water content and solubility (although not significantly different from the control), which may be related to its enhanced susceptibility to microbial activity. Increased water content and solubility can facilitate microbial colonization and enzymatic hydrolysis, thereby accelerating biodegradation. However, the differences between the samples in the biodegradation test were not statistically significant, suggesting that all the developed composites exhibit a similar, favorable level of biodegradability [3,57].

Eco-toxicity analysis using cress (*Lepidium sativum* L.) germination showed that aqueous extracts of films containing plant extracts (BB and SAGE) caused a decrease in respiratory activity (RA8) to 66.9 ± 4.2 and 59.4 ± 5.2 mg O_2_/100 seeds, respectively. Compared to the control film (114.6 ± 10.2 mg O_2_/100 seeds), this indicates a potential impact of bioactive components on the metabolism of germinating plants. This effect may result from the antibacterial action of the extracts used and does not necessarily indicate toxicity, but rather a change in seed germination kinetics [58].

Biodegradation of organic matter can occur at different rates depending on the process conditions (air availability, temperature) and the additives introduced into the matrix. The degree of its polymerization changes significantly. More complex humic acid molecules are formed, while the content of simpler fulvic acid molecules decreases. These fractions may vary in terms of molecular size, chemical composition (including the complexity of structural branching and the content of redox-active functional groups), as well as properties (e.g., aromaticity, hydrophobicity, polarity and polyelectrolyte nature) [59].

The favorable level of biodegradability demonstrated in respiratory studies, regardless of the composite used, was also confirmed in the analysis of the fractional composition of humic substances (Table 7).

The analysis of the humic substances’ fractional composition in vermicompost with the addition of BB and SAGE showed a significant increase in extractable carbon content and humic acid carbon compared to the control vermicompost. The beneficial effect of adding BB and SAGE to the vermicompost was also reflected in the value of the C_HA: C_FA ratio, which was 20.7% (BB addition) and 5.1% (SAGE addition) higher, respectively, compared to the control vermicompost. The obtained results demonstrate the beneficial influence of BB and SAGE on the transformation of humic substances in vermicompost, which, under soil application conditions, act as biologically active substances that positively affect plant physiology by improving soil structure and fertility, as well as by influencing nutrient uptake and root architecture [60].

## 3. Materials and Methods

### 3.1. Materials Used for Active Double-Layer Film Preparation

The following biopolymers were used for the development of active bilayer films: κ-carrageenan, porcine gelatin and carboxymethyl cellulose, all purchased from Pol-Aura (Morąg, Poland). Sage flowers were obtained from Plantago (Działdowo, Poland), while blackberry fruits were sourced from HiFOOD (Kolbuszowa, Poland). Glycerol was supplied by Chemland (Stargard, Poland).

### 3.2. Preparation of Double-Layer Active Films

The film-forming solution was prepared by dissolving carrageenan in distilled water to obtain a 2% (*w*/*v*) solution using a magnetic stirrer with heating function (Heidolph, Schwabach, Germany). Subsequently, porcine gelatin was incorporated into the solution at a weight ratio of 2:1 (gelatin to carrageenan).

Aqueous plant extracts were prepared using dried sage and blackberry fruit. Preparation of 10% *w*/*v* aqueous extracts was carried out by weighing the plant material and adding deionized water, then placing them on a magnetic stirrer (Heidolph, Poland) for 30 min under controlled extraction temperature conditions of 70 °C. After 30 min, the extract was filtered through Whatman No. 1 filter paper to obtain a clear solution. Aqueous extracts of sage and blackberry fruits were then added at 12.5% of the total volume of the film-forming layer.

The second layer comprised an equal volume of a 2% (*w*/*v*) carboxymethyl cellulose (CMC) film-forming solution. Glycerol was used as a plasticizer at a concentration of 1 g per 100 mL of the solution. The film-forming solutions were poured onto trays using a layer-by-layer casting method.

The preparation of double-layer films was based on the ability of the first layer forming the film to develop a stable gel structure. After preparing the film-forming solution for the first layer, it was poured onto a tray placed under a laboratory fume hood. The layer was left until it reached a hard gel consistency, ensuring structural stability and preventing the layers from mixing. Then, a second layer was poured onto the surface of the gel layer, and the entire system was left to dry under the laboratory fume hood.

Three types of films were developed: one containing sage extract (SAGE), one comprising blackberry extract (BB), and a control film (control) composed solely of the biopolymer matrix. No intentional covalent crosslinking agents were used.

### 3.3. Physicochemical and Structural Characterization of Films

#### 3.3.1. FTIR Spectroscopy

The IR analysis of films was performed in accordance with the methodology described previously [61]. To account for the layered nature of the films, infrared analysis was carried out separately for the first and third layers of the multilayer structure. The spectra were collected using the Nicolet iS5 FTIR spectrometer (Thermo Fisher Scientific, Waltham, MA, USA), operating within the range of 4000 to 700 cm^−1^. The thickness of the composites was determined using a Mitutoyo No. 7327 manual micrometer (Kawasaki, Japan).

#### 3.3.2. UV–Vis Spectroscopy Analysis

Analysis of UV light blocking ability was performed using the Shimadzu 2101 spectrophotometer (Shimadzu, Kyoto, Japan) in the wavelength range of 300–700 nm. Opacity was measured at 660 nm according to the following equation:

(1) $$Opacity=\;A_{660\;}÷\;X$$

A_600_—absorbance value at 660 nm; X—film thickness [mm].

The analysis was performed in accordance with the methodology described previously, with modifications [61].

#### 3.3.3. Water Vapor Transmission Rate (WVTR)

Water vapor transmission rate (WVTR) was assessed following the ISO 2528:2017 [62] standard. The test involved sealing a dish containing silica gel with the film samples and placing it in a controlled-environment climatic chamber. Measurements were conducted at 25 °C and 75% relative humidity.

The water vapor transmission rate (WVTR) was calculated using the following formula:

(2) $$\mathrm{WVTR}\;(g\;\times \;m^{-2}\;\times \;d)\;=\;240\;\times \;(\frac{water\;weight}{surface\;penetration})\;\times 24$$

#### 3.3.4. Water Content and Solubility

Water-related parameters were assessed based on the procedures described by Pastor et al. [63] and Irissin-Mangata et al. [64]. Prior to analysis, the film samples were stored in a desiccator (relative humidity ≈ 53%) for 48 h to allow for moisture equilibration.

Square sections of the films (20 × 20 mm) were initially weighed to the nearest 0.0001 g to obtain their initial mass (W1). These were then dried at 70 °C for 24 h, after which the dry mass (W2) was recorded. The dried films were immersed in 30 mL of deionized water and kept at room temperature for another 24 h.

Following the soaking period, samples were removed, carefully dried again at room temperature for 24 h, and weighed to determine the final mass of the insoluble fraction (W3).

The following equations were applied to calculate the water content and solubility of the films:

(3) $$Water\;content\;\left[\%\right]=[\left(W_{1}-\;W_{2}\right)\times 100]\;÷\;W_{2}$$

(4) $$Solubility\;[\%]=\left[\left(W_{1}-W_{3}\right)\times 100\right]÷\;W_{1}$$

#### 3.3.5. Mechanical Properties

Mechanical testing was carried out according to the EN ISO 527 standard [65] (test method D882–02), with focus on evaluating tensile strength (TS), elongation at break (EAB), maximum breaking load (MBL) and Young’s modulus. Prior to analysis, the films were cut into rectangular strips (300 × 15 mm) using a standardized cutter to minimize variability caused by uneven sample edges. The samples were conditioned for 24 h at 23 °C and 50% relative humidity.

Measurements were performed using a tensile testing system at a grip separation speed of 25 mm·min^−1^. Tensile parameters were calculated from the resulting stress–strain curves, obtained during the stretching of each sample under controlled conditions.

### 3.4. Bioactive Properties of Films

#### 3.4.1. Analysis of Antioxidant Properties

The antioxidant potential of the film was analyzed using the Fe^3+^ iron ion reduction ability (FRAP) in accordance with the method proposed by Khantaphant and Benjakul [66], the metal chelating activity (according to the method proposed by Xie et al. [67]), and the DPPH free radical scavenging activity was determined using the method proposed by Wu et al. [68].

Initially, a FRAP solution was prepared that contained an acetate buffer (pH 3.6), ferric chloride solution (20 mM) and a solution of 2,4,6-tripyridyl-s-triazine in hydrochloric acid (10 mM TPTZ in 40 mM HCl) at a volume ratio of 10:1:1. The solution prepared was incubated in the dark for 30 min at 37 °C. Film extracts (15 mg/mL) were also prepared by mixing 150 mg of each analyzed film with 10 mL of distilled water (at 45 °C). The mixture was then heated and shaken in a water bath at 50 degrees for 10 min to dissolve the films.

At that point, the film extracts prepared in this way were incubated with the prematurely prepared FRAP reagent for 10 min at 37 °C. The components were mixed at a volume ratio of 0.4:3.6. After incubation, absorbance was measured using the Helios Gamma UV-1601 spectrophotometer (Thermo Fisher Scientific, Waltham, MA, USA) at 593 nm.

The ability of the film to chelate iron ions was performed by extracting 1 mL of the film with 50 µL of FeCl2 (2 mM) and 1.85 mL of distilled water. Then, 100 µL of 5 mM ferrozine was added and mixed thoroughly. The mixture was allowed to stand at room temperature for 10 min. After this time, absorbances were determined at 562 nm. In the blank sample, 1 mL of the film extract was replaced with 1 mL of distilled water. The results were expressed as % of metal ion chelating capacity.

Analysis of DPPH free radical scavenging capacity began by mixing the film extract (at a concentration of 75 mg/mL) with a 0.1 mM solution of 2,2-diphenyl-1-picrylhydrazyl in ethanol at a volume ratio of 0.2:2.8. The resulting solution was then incubated for 10 min at room temperature. After this time, absorbances were measured at 517 nm using a spectrophotometer (Thermo Fisher Scientific, Waltham, MA, USA). In the blank sample, 1 mL of the film extract was replaced with 1 mL of distilled water. The results were expressed as % ability to deactivate the DPPH radical.

#### 3.4.2. Antimicrobial Properties of Films

Antibacterial properties were tested according to the method proposed by Grzebieniarz et al. [69]. Microbiological analysis of the materials was performed with regard to the following microorganisms: *Candida albicans* ATCC 10231, *Candida krusei* ATCC 6258, *Aspergillus brasiliensis* ATCC 204304, *Aspergillus flavus* ATCC 16404, *Escherichia coli* ATCC 25923, *Enterococcus faecalis* ATCC 29212, *Pseudomonas aeruginosa* ATCC 27869, *Staphylococcus aureus* ATCC 25922 and *Salmonella enterica* BAA664. The bacterial suspension was prepared to a standard of 0.5 on the McFarland scale and spread evenly on a Petri dish (⌀90 mm) containing Müller-Hinton agar (for bacteria) or Sabouraud agar supplemented with glucose (for yeast). Circular film samples with a diameter of 5 mm were cut out and sterilized using a UV lamp. Under sterile conditions, they were placed on agar plates and incubated at 37 °C for 24 h. The growth of microorganisms in the areas surrounding and covering the films was assessed visually.

### 3.5. Packaging Performance Evaluation—Experimental Setup for Salmon Storage

#### 3.5.1. Experimental Setup for Salmon Storage

Atlantic salmon (*Salmo salar*) fillets were purchased from a wholesale retail store (Makro, Krakow, Poland) on the day of the experiment. The fillets were portioned into smaller samples of approximately 100 ± 2 g each, kept under aseptic conditions and individually wrapped in the prepared films. A control group was wrapped in a low-density polyethylene (LDPE) film. All samples were stored at 4 °C in the dark and analyzed at defined intervals: on the day of packaging (D0) and after 3, 6, 9 and 12 days (D3, D6, D9, D12, respectively).

Each time point included three biological replicates per treatment group. The following parameters were evaluated: total viable microbial count, counts of yeasts and molds, psychrotrophic bacteria, lipid oxidation using the TBARS assay and pH of the fish muscle.

#### 3.5.2. Microbiological Analyses of Atlantic Salmon

Microbiological analyses of Atlantic salmon samples were performed according to standard protocols for food testing. Sample preparation for microbial enumeration followed the guidelines outlined in ISO 6887-1:2017 [70]. Total viable counts (TVC) were determined using the pour plate technique on the Plate Count Agar (Biomaxima, Warsaw, Poland), as recommended in ISO 4833-1:2013 [71]. The plates were incubated at 30 °C for 48 h. Psychrotrophic bacterial counts were assessed using the same plating method, with incubation extended to 240 h at 6.5 °C, following ISO 17410:2019 [72]. Yeasts and molds were enumerated by surface spreading on a dichloran rose bengal chloramphenicol (DRBC) agar (Biomaxima, Warsaw, Poland). These samples were incubated at 25 °C for 120 h.

#### 3.5.3. Thiobarbituric Acid Reactive Substances (TBARS)

To assess the level of lipid oxidation, TBARS analysis was performed on salmon samples stored in experimental films. On the corresponding days of analysis, a sample of salmon stored in the analyzed films was taken and homogenized. Subsequently, 10 g of the homogenate were taken and mixed with 30 mL of 4% perchloric acid. Then, 4% ethanolic solution of butylated hydroxytoluene (BHT) in the amount of 0.75 mL was added to the mixture. The mixture was again homogenized. Following this step, it was centrifuged at 10.733× *g* for 10 min and filtered into 50 mL volumetric flasks through Whatman blotting paper No. 1. The filtrate was supplemented with perchloric acid to achieve the final volume. The contents of the flask were thoroughly mixed, and 4 mL of the solution was transferred and mixed with the same volume (4 mL) of 0.02 M thiobarbituric acid (TBA) solution. The reaction mixture was incubated in a water bath at 90 °C for 1 h. After incubation, the samples were allowed to cool at room temperature, and the absorbance at 532 nm was measured using the Helios Gamma spectrophotometer (Thermo Fisher Scientific, Essex, UK). A blank sample was prepared using 4% perchloric acid instead of the homogenate.

#### 3.5.4. pH Measurement

The pH of the samples was measured by homogenizing the salmon samples and then thoroughly mixing 5 g of the homogenizer with 5 g of distilled water. The pH was measured using the CP 505 pH-meter (Elmetron, Zabrze, Poland).

### 3.6. Assessment of Environmental Impact

#### 3.6.1. Biodegradation Assessment of Films

Analyses of the biodegradation and respiratory activity of the films were carried out according to the methodology published by Tkaczewska, Jamróz, Guzik and Kopeć [57], with modifications. In order to test the degree of biodegradation, double-layered films were incubated based on the methodology proposed by the International Organization for Standardization (ISO), number 14855–1:2012 [73]. The respiratory activity of the material during stimulated biological changes (composting) was determined under controlled composting conditions, in accordance with the methodology described by Tkaczewska, Jamróz, Guzik and Kopeć [57], with alterations. Briefly, 1 g (dry weight) of the tested film was placed in 9 g of the vermicompost and left for incubation (28 days, 45 ± 1 °C). The incubation of the films was conducted in vessels with a 2.5 dm^3^ capacity. The discussed analyses were performed in duplicate.

The manometric measurement of respiration activity was applied via Oxi-Top measuring equipment (WTW, Munich, Germany). The recorded pressure-related changes were directly proportional to the amount of oxygen absorbed by the sample, which was the result of breathing processes that naturally occur in the sample (OxiTop^®^ 2003). The pressure changes were noted every 60 min. The amounts of the resultant CO_2_ equivalent were then absorbed by the 1 mol × dm^−3^ NaOH solution, which was contained in the vessels. The respiratory activity of the materials was converted to dry weight.

The assessment of the biodegradation efficiency of the materials was also related to the fractional composition of humic compounds. The fractional organic matter composition of the materials was determined according to the Schnitzer method (Griffith and Schnitzer 1975 [74]). Humic compounds were extracted from the materials using a solution of 0.5 mol·dm^−3^ NaOH. In the alkaline extract, the carbon content of fulvic acids (Ckf) was determined after precipitation using H_2_SO_4_ at a concentration of 2 mol·dm^−3^ of humic acids (Ckh), which were separated via suspension filtration. The carbon content of humic acids (Ckh) was calculated from the difference between the C content of the extract and the C content of fulvic acids. The ratio of Ckh to Ckf was calculated as the quotient of the C content for the two fractions. The tests were performed in triplicate [74].

#### 3.6.2. Ecotoxicity Testing

Analysis of ecotoxicity was carried out under closed-vessel conditions by evaluating the growth of cress (*Lepidium sativum* L.) in contact with an aqueous film extract. The 100-seed weight was 0.258 ± 0.002 g. The degree of toxicity was estimated as the resultant CO_2_ (measured using the OxiTop system), with respect to the object placed in distilled water. These analyses were performed in duplicate.

### 3.7. Statistical Analysis

All data are presented as mean values ± standard deviation (SD), with a minimum of three replicates per treatment group (n ≥ 3). Prior to statistical evaluation, normality of data distribution was verified using the Shapiro–Wilk test. All statistical analyses were performed using Statistica software (version 13.0, Tibco Software Inc., Palo Alto, CA, USA).

The results obtained from film property measurements were analyzed using one-way analysis of variance (ANOVA), whereas data related to the salmon storage study were subjected to two-way ANOVA, taking into account both storage duration and film type as independent variables. Group means were compared using Tukey’s post hoc test, with statistical significance set at *p* ≤ 0.05.

## 4. Conclusions

The conducted analyses showed that the incorporation of a double-layer protein–polysaccharide matrix enriched with antimicrobial plant extracts enabled the functionalization of biocomposites and enhanced their performance characteristics as well as their applicative potential. The resulting films exhibited strong UV-blocking capability and moderate water vapor permeability, which remained stable despite the incorporation of aqueous extracts. Although a reduction in tensile strength was observed, the materials displayed increased elasticity and extensibility, suggesting a reorganization of the biopolymer network induced by phenolic compounds in the extracts, which acted as natural plasticizers. Microbiological evaluation revealed that the active films substantially inhibited the growth of aerobic microflora, psychrotrophs, yeasts and molds in stored salmon, particularly on days 6 and 9 of storage. Nevertheless, the films accelerated lipid oxidation, indicating a need for further optimization. Biodegradation studies confirmed the high respiratory activity of all tested composites under composting conditions, while phytotoxicity assays indicated reduced seed germination activity, likely due to the antimicrobial properties of the extracts. Overall, the results highlight the potential of plant extract-enriched biocomposites not only to enhance material performance but also to support sustainable waste management and soil fertility when applied under environmental conditions.

## Figures and Tables

**Table 1:** Water properties of biocomposites.

Type of Film/Parameter	Water Content [%]	Solubility [%]	WVTR [g/m^2^ × d]
Control	7.89 ± 0.38 ^a^	53.45 ± 1.26 ^a^	543.02 ± 17.64 ^a^
BB	9.54 ± 1.39 ^b^	41.73 ± 1.73 ^b^	588.62 ± 33.07 ^a^
SAGE	10.67 ± 0.26 ^ab^	55.99 ± 1.14 ^a^	548.49 ± 38.06 ^a^

^a,b^ different letters indicate significant differences between measures.

**Table 2:** Mechanical properties of biocomposites.

Type of Film/Parameter	Max Breaking Load [N]	Tensile Strength [MPa]	Elongation at Break [%]	Young’s Modulus [N/mm^2^]
Control	83.09 ± 4.97 ^c^	5.54 ± 0.33 ^c^	20.00 ± 2.42 ^a^	477.58 ± 50.03 ^b^
BB	51.92 ± 2.87 ^b^	3.46 ± 0.19 ^b^	35.35 ± 2.11 ^b^	127.33 ± 24.74 ^a^
SAGE	38.27 ± 0.96 ^a^	2.55 ± 0.06 ^a^	39.29 ± 0.84 ^b^	103.23 ± 7.56 ^a^

^a,b,c^ different letters indicate significant differences between measures.

**Table 3:** Antioxidant properties of films.

Type of Film/Parameter	FRAP [µTrolox/mg of Dried Films]	DPPH [%]	Metal Chelating Activity [%]
Control	0.38 ± 0.22 ^a^	6.38 ± 0.74 ^a^	0 ^a^
BB	1.97 ± 0.17 ^b^	85.68 ± 8.25 ^b^	0 ^a^
SAGE	4.48 ± 0.23 ^c^	78.25 ± 0.23 ^b^	0 ^a^

^a,b,c^ different letters indicate significant differences between measures.

**Table 4:** Zone [mm] of microbial growth inhibition for analyzed films.

Microorganism	Control	BB	SAGE
*Candida albicans*	No effect	No effect	No effect
*Candida krusei*	No effect	No effect	No effect
*Aspergillus brasiliensis*	No effect	No effect	No effect
*Aspergillus flavus*	20 ± 0.0	20 ± 0.0	20 ± 0.0
*Escherichia coli*	No effect	No effect	No effect
*Enterococcus faecalis*	No effect	No effect	No effect
*Pseudomonas aeruginosa*	No effect	No effect	No effect
*Staphylococcus aureus*	No effect	No effect	No effect
*Salmonella enterica*	No effect	No effect	No effect

**Table 5:** Changes in pH level and TBARS [mg MDA/kg] for salmon during storage.

**TBARS [mg MDA/kg]**					
Type of Film/Day	D0	D3	D6	D9	D12
Control	0.08 ± 0.03 ^A^	0.14 ± 0.03 ^aA^	0.66 ± 0.27 ^aBC^	0.51 ± 0.21 ^aB^	0.77 ± 0.18 ^aC^
BB		0.21 ± 0.02 ^bB^	0.64 ± 0.21 ^aCE^	0.92 ± 0.19 ^cD^	0.63 ± 0.19 ^aEC^
SAGE		0.19 ± 0.04 ^bA^	0.41 ± 0.19 ^abB^	0.51 ± 0.18 ^aBC^	0.63 ± 0.10 ^aC^
LDPE		0.19 ± 0.01 ^aB^	0.30 ± 0.05 ^bC^	0.30 ± 0.05 ^bC^	0.28 ± 0.05 ^bC^
pH					
Control	6.34 ± 0.06 ^A^	6.35 ± 0.06 ^aA^	6.30 ± 0.13 ^aBAD^	6.60 ± 0.16 ^aCA^	6.27 ± 0.11 ^aDAB^
BB		6.31 ± 0.06 ^aAB^	6.34 ± 0.08 ^aAB^	6.28 ± 0.08 ^bAB^	6.21 ± 0.03 ^aB^
SAGE		6.32 ± 0.04 ^aA^	6.30 ± 0.04 ^aA^	6.32 ± 0.03 ^bA^	6.30 ± 0.09 ^aA^
LDPE		6.38 ± 0.09 ^aA^	6.39 ± 0.12 ^aA^	6.36 ± 0.07 ^abA^	6.73 ± 0.04 ^bB^

^a,b,c^ different letters indicate significant differences between measures. Uppercase letters indicate differences in the same film over time, and lowercase letters indicate differences between films.

**Table 6:** Respiratory activity equations in the study, biodegradation and phytotoxicity of biocomposites.

Respiratory Activity			
	Control	BB	SAGE
1–48 h			
y	1.7291x − 1.4105	1.6714x − 2.6477	1.6881x + 1.304
R^2^	0.9453	0.9353	0.9496
49–672 h			
y	0.0994x + 78.636	0.1047x + 71.41	0.1123x + 83.335
R^2^	0.984	0.985	0.991
RA_28_ (mg O_2_/g d.m.)	140.9 ± 8.8 ^a^	137.2 ± 7.4 ^a^	156.3 ± 10.1 ^a^
Toxicity test			
RA_8_ [mg O_2_ × (100 seeds)^−1^]	114.6 ± 10.2 ^a^	66.9 ± 4.2 ^b^	59.4 ± 5.2 ^b^

Values in rows marked with the same letters do not significantly differ (*p* ≤ 0.05). RAx—Respiratory activity accumulated oxygen uptake over a period of x days (mg O_2_/g d.m.). R^2^—coefficient of determination. y = ax + b, where x—time [h]; [mg O_2_/g d.m.].

**Table 7:** Fractional composition of humic compounds [g·kg^−1^ d.m.].

Type of Film/Day	C Extract	C Humic Acids	C Fulvic Acids	Ckh/Ckf Ratio
Control	73.3 ± 1.3 ^a^	31.8 ± 0.3 ^a^	41.5 ± 2.3 ^b^	0.77 ± 0.18 ^a^
BB	78.0 ± 1.1 ^c^	37.6 ± 2.5 ^c^	40.3 ± 0.6 ^a^	0.93 ± 0.86 ^c^
SAGE	75.8 ± 1.6 ^b^	34.0 ± 3.1 ^b^	41.8 ± 1.51 ^b^	0.81 ± 0.61 ^b^

^a,b,c^ different letters indicate significant differences between films.

## Data Availability

The original contributions presented in this study are included in the article. Further inquiries can be directed to the corresponding author.
